# Practical and environment-friendly indirect electrochemical reduction of indigo and dyeing

**DOI:** 10.1038/s41598-020-61795-5

**Published:** 2020-03-18

**Authors:** Changhai Yi, Xiaodong Tan, Bihan Bie, Haitao Ma, Hong Yi

**Affiliations:** 1 Science and Technology Institute, National Engineering Laboratory for Advanced Yarn and Fabric Formation and Clean Production, Wuhan Textile University, Wuhan, Hubei 430200 People’s Republic of China; 2 College of Chemistry and Molecular Sciences, Wuhan University, Wuhan, Hubei 430072 People’s Republic of China; 3 Department of Material Engineering, Faculty of Textile Engineering, Technical University of Liberec, Liberec, Czech Republic; 4 Shandong Ruyi Technology Group Company, Jining, Shandong People’s Republic of China

**Keywords:** Electrocatalysis, Environmental monitoring

## Abstract

Indigo has been widely used as a dye in the industrial dyeing due to its good color fastness in dyeing cellulose fibers. However, excess reducing agent, “insurance powder (Na_2_S_2_O_4_)”, was always used in the actual production of the factory, sparking serious pollution (water pollution and air pollution). Herein, we developed a practical and environment-friendly indirect electrochemical reduction of indigo, and applied this method for cloth dyeing. The electrochemical device was designed in the combination of source of electro-catalytic reduction and dyeing. The iron-triethanolamine-calcium gluconate (Fe-TEOA-Ca) complex played a role of key intermediate, and ultrasonic wave was found to speed up the indirect electro-catalytic process. The electrochemical performance of intermedia was improved by calcium ion addition. Washed with oxalic acid solution, the dyed fabric could achieve the level of color fastness in industry standard. Generally speaking, our method leads to a green route for indigo reduction using electrochemistry, which may change the crafting process of indigo dyeing in industry.

## Introduction

**Figure 1:**
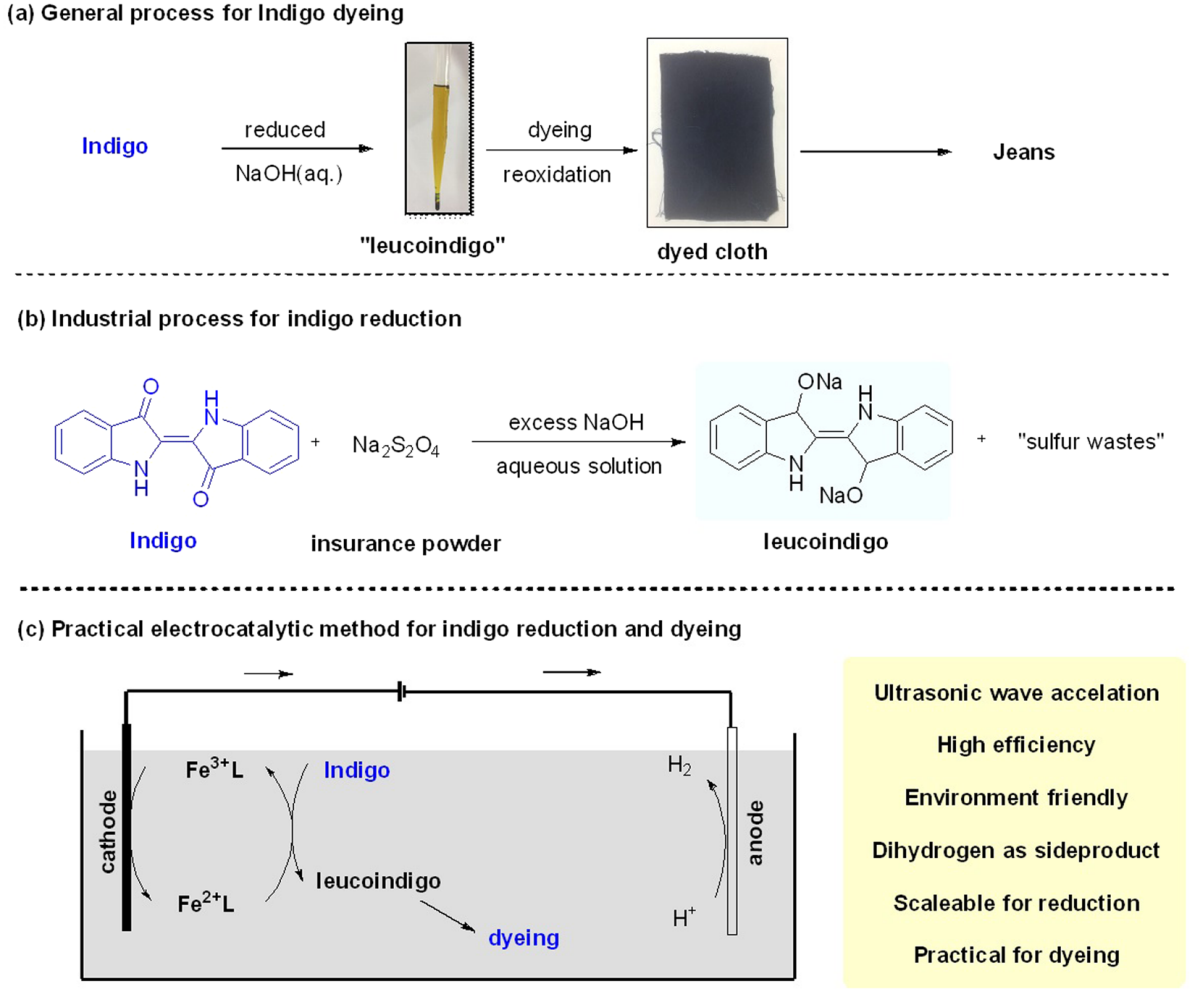
Electrocatalytic indigo reduction. (**a**) General process for indigo dyeing; (**b**) Industrial process for indigo reduction; (**c**) Practical electrocatalytic method for indigo reduction and dyeing.

In the dyeing process of cellulose fibers, indigo dyes mostly hold the market share of dyes. Indigo has shown great application in the dyeing industry due to its good color fastness in dyeing cellulose fibers, especially in the light resistance, water washing and chlorine bleaching process^1,2^. However, due to its water insolubility, some technical problems are staring at indigo. In the dyeing process (Fig. 1a), indigo needs firstly to be reduced to a water-soluble leuco indigo. In this reduced form, leuco indigo has a direct affinity with cellulose fibers. After dyeing to the fiber, the leuco indigo on the cellulose fibers is oxidized by air to regenerate the insoluble indigo, finally achieving the dyeing process. The dyed cloths can be further used for clothes-making^3^. In this process, reduction of indigo to leuco indigo is the initial and important step in the dyeing process. Sodium dithionite (Na_2_S_2_O_4_), called “insurance powder”, is a common-used reducing agent to reduce indigo in the industry (Fig. 1b). Although “insurance powder” has reams of advantages including cheap, widely-available and easy-to handle and so on. To date, some problems over pollution is a common sight during indigo dyeing. After the reduction of indigo dye by excess “insurance powder”, several sulfur-related side produces such as sulfites, sulfates and toxic sulphides, are brought about at the same time, causing serious water pollution and air pollution^4^. In order to quench current desire for friendly environment, several reducing agents such as α-hydroxyl ketones, hydroxyalkyl sulfonate and other strategies have been developed instead of sodium hyposulfite^5–10^. However, the poor reductive ability and high cost of those agents strand the further application in the practical industry. Therefore, the development of a practical method to replace excess reducing agent is imminent.

For ecological and economic reasons, electrochemical reduction is an attractive technology to replace the traditional reduction method using excess reductants^11^. In this electro-chemical method, the reduction of indigo dyes can be given by the transfer of electrons with electricity, and dihydrogen (H_2_) as the side product. In recent years, the development of electrochemical reduction of indigo has been sent to the spotlight including direct electro-chemical reduction and indirect electro-chemical reduction^12,13^. Although direct electro-chemical reduction leads to a straightforward way for indigo reduction, while the efficiency is too poor to satisfy the practical use of industry as the mass-transfer between indigo solution and cathodes^14,15^. The indirect electro-chemical reduction by using a media to reduce the indigo can address the mass transfer and improve the reductive efficiency^16–18^. The intermediate mediates have been optimized by measuring current efficiency and electrochemical parameters, in which iron salt is proved to be an efficient media to reduce indigo^18–28^. Although several reports existed, the low concentration reductive leuco indigo can not be used for dyeing, and also dyeing fails to be realized in the combination of electrochemical indigo reduction. In this work, we developed a practical and environment-friendly indirect electrochemical reduction of indigo, and applied this method to cloth dyeing (Fig. 1c). Iron-triethanolamine-calcium gluconate (Fe-TEOA-Ca)^29^ was used as a novel binuclear complex system. As ultrasonic wave comes out, the indirect electrochemical reduction of indigo dye was carried out, and the electrode process, current efficiency, polarization curve and dyeing effect of the reduction were also studied and discussed. After washing with oxalic acid solution, the dyed fabric could achieve the level of color fastness in industry standard. This method provides a green route for indigo reduction and dyeing with indirect electrochemistry.

## Results

### Concept and design of electrochemical dyeing device

**Figure 2:**
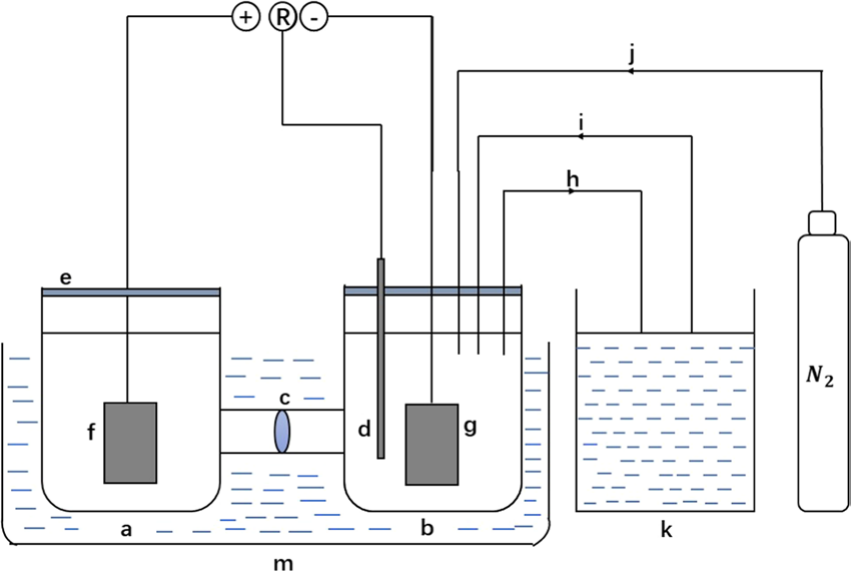
Device of electrochemical reduction and dyeing ((**a**) anode chamber; (**b**) cathode chamber; (**c**) ion exchange membrane; (**d**) reference electrode; (**e**): entry board; (**f**) anode electrode; (**g**) cathode electrode; (**h**) water pump; (**i**) water pump; (**j**) tube; (**m**) ultrasonic tank; (**k**) dyeing pool).

At first, the device of electrochemical reduction and dyeing was designed to achieve electrolysis and dyeing in one device (Fig. 2). The electrolysis experiment was carried out in this device with constant current mode. The cathode chamber and the anode chamber were separated by membrane. Both cathode and anode were employed the stainless steel or nickel electrode (4*5 cm^2^), while the saturated calomel electrode (SCE) played a role of a reference electrode. The volume of both electrolyte was 300 mL using Ca^2+^-Fe^3+^-TEOA or Fe^3+^-TEOA mediator solution including indigo and 40 g/L NaOH solution as catholyte and anolyte, respectively. Catholyte was stirred by mixer and ultrasound wave was used during electrochemical reaction experiment under nitrogen atmosphere. The electrochemical reaction duration was 2 hours. After the complement of electrochemical reduction, the catholyte was transferred to dyeing tank for dyeing by a water pump. During the dyeing spans, the oxidized liquid was conveyed to cathode tank simultaneously achieving the recycle of catholyte. This device combines the process of electrolysis and dyeing, which could achieve the integration and the recycling of dyes (Figure S1).

### Investigation of current efficiency

**Figure 3:**
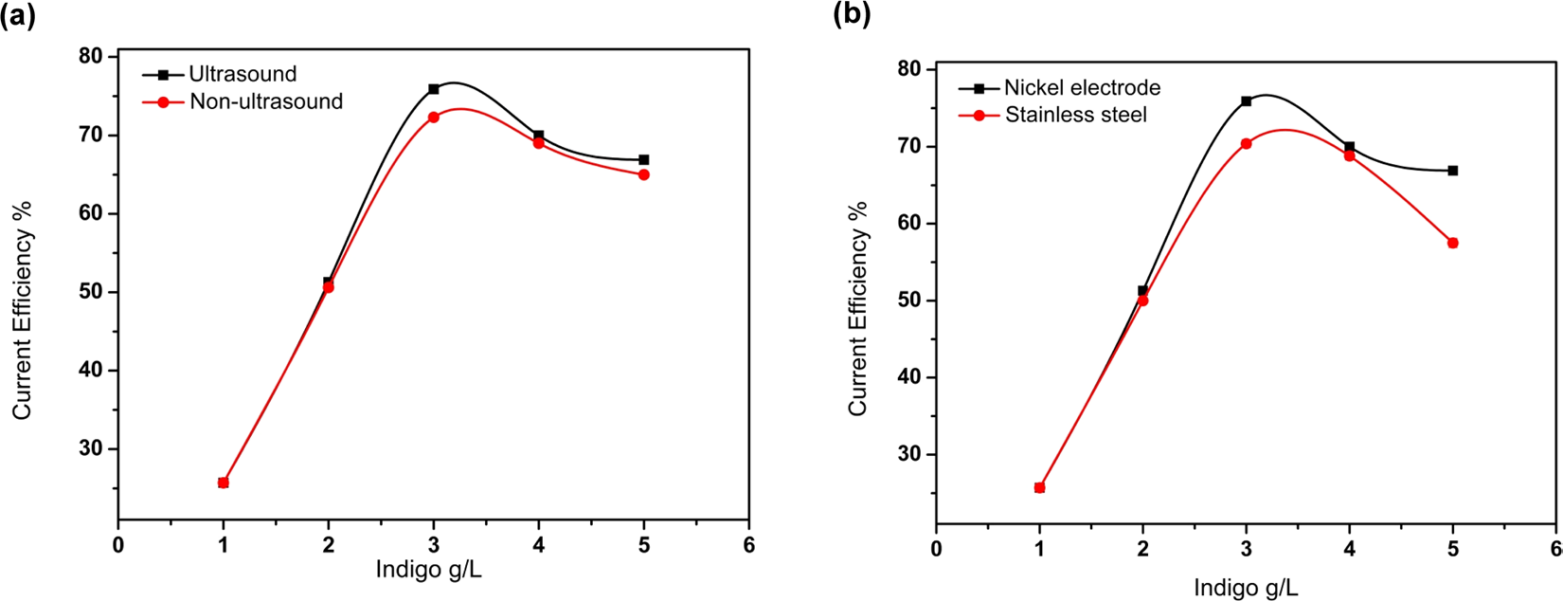
Investigation of current efficiency.

The materials of electrodes and the external condition would remarkable bear on the current efficiency (CE) of electrochemical reduction. Therefore, we investigated the effect on the current efficiency of the ultrasonic and electrodes independently (Fig. 3). With the increase of indigo concentration, the CE of electrochemical reduction under ultrasonic conditions is generally steeper than that in the absence of ultrasonic wave (Fig. 3a), of which an explanation is that ultrasonic wave may accelerate the mass transfer process of the solution^30^. In addition, the cavitation of the ultrasonic wave trigger impacting, washing and peeling effects on the electrode, and in this way, the surface of the cathode is cleaned and deposits of the solution medium on the electrode surface is reduced, which could cement the electrochemical activity of the electrode (Figure S2, iron deposition on the cathode surface). The polarization curves of different electrodes were then investigated (Figure S3, polarization curve of different electrodes). During the electrochemical reaction, the CE value of nickel is higher than that of stainless steel (Fig. 3b). The reason is that the nickel electrode has catalytic property, which speeds up the efficiency of reducing indigos. Also, the nickel electrode has better corrosion resistance and electrochemical activity than the stainless steel. Over time, the nickel electrode maintains a high rate of reduction, while the CE of the stainless steel is affected by deposits and corrosion.

### Cyclic voltammetry (CV) experiments of mediators

**Figure 4:**
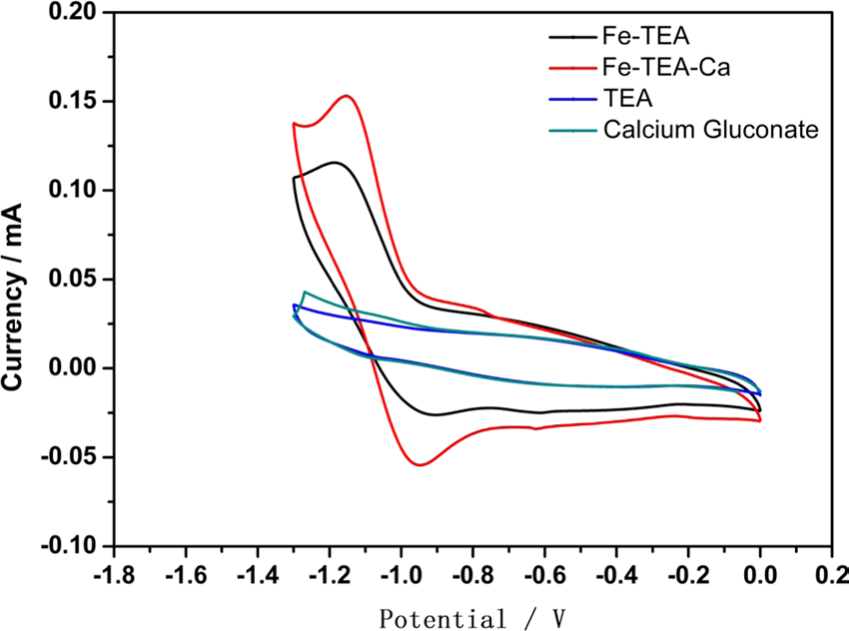
Cyclic voltammetry of different mediators.

Choosing a suitable mediator is crucial for the indirect electrochemical process, which can make it easy to reduce the indigo dye and improve the reduction efficiency. As shown in Fig. 4, the redox peak of the pure TEA indigo solution and pure calcium gluconate indigo solution did not occur when scanned with cyclic voltammetry curve. However, there are remarkable oxidation peaks and reduction peaks in the Fe-TEOA and Fe-TEOA-Ca system in the cyclic voltammetry curve. Furthermore, the reduction peak current and oxidation peak current of the Fe-TEOA-Ca system are significantly higher than that of the Fe-TEOA system. Its oxidation peak potential shifts to negative potential and the reduction peak potential shifts to positive potential. This stems from to an extend the addition of Ca^2+^ ions, which combines with Fe to form a Fe-TEOA-Ca binuclear system. Adding Ca^2+^ ions, it improves the electrochemical activity of the intermedia. This entire complex system group carries more charges, which may enhance the electrostatic attraction and the ability of transfer of electrons.

### Investigation the best conditions

**Figure 5:**
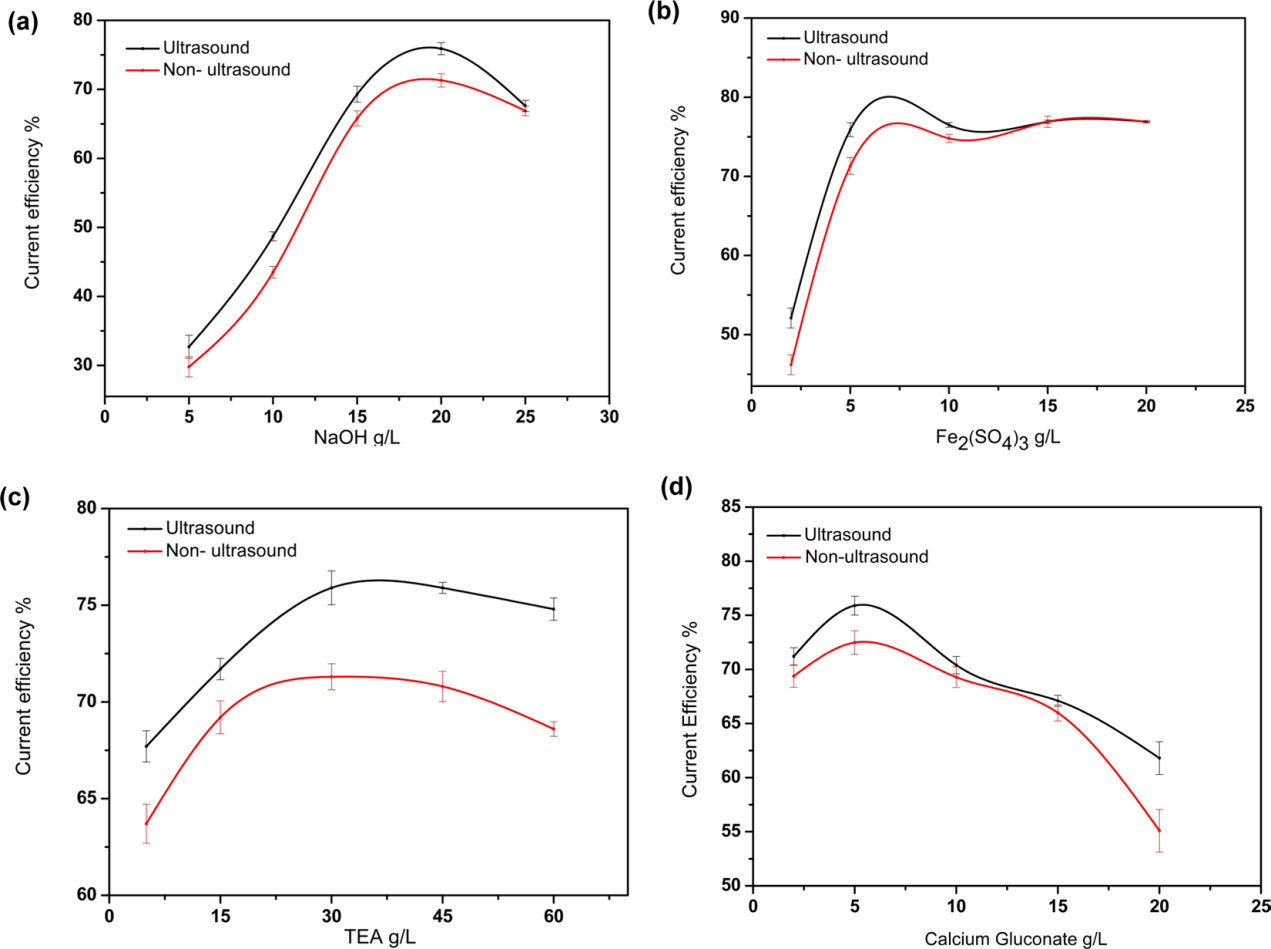
Investigation the reaction conditions.

Using Fe-TEA-Ca binuclear system as the mediator, we then investigated different paraments by testing its CE and K/S, which are usually used to judge the color of the fabric. Both conditions under ultrasound and non-ultrasound were investigated. When NaOH concentration was low, the PH value of the solution could not achieve the conditions, in which indigo is reduced to leuco indigo compound, with poor reduction efficiency and current efficiency (Fig. 5a). As shown in Fig. 5b, the CE increased as the concentration of ferric sulfate increased. With the increase of TEA concentration, the reduction efficiency and current efficiency increase slightly and then decrease a little (Fig. 5c). The CE value reached its peak when TEA concentration was 30–40 g/L. When the concentration of calcium gluconate is 5 g/L, the reduction efficiency and current efficiency reached the maximum (Fig. 5d). The investigation results revealed that the best reduction temperature should be 50 °C (Figure S4, investigation of pool temperature). The reason is that more negative charges are afforded when the concentration of calcium gluconate rises, further increasing electrostatic attraction. In addition, all the CE with ultrasound was higher than that without ultrasound. Overall, the CE and the K/S of Fe-TEA-Ca system with current density of 1.0 A/dm^2^, NaOH concentration of 20.0 g/L, ferric sulfate concentration of 5.0 g/L, TEA concentration of 30.0 g/L, calcium gluconate concentration of 5.0 g/L, reduction temperature of 50°C, reduction time of 2.0 h could achieve the best CE value.

### Dyeing process

The dyeing process using reduction solution by electrochemical process came as the achievement of the best conditions. After dyeing with cotton fabrics, we found that many particles deposited on the surface of fabrics and the color of fabrics and a subtle nuance between the color of fabrics and traditional denims (Figure S5, scanning image of pure white cotton cloth and traditional indigo dyed cloth), which may stem from some iron oxide attaching on the fabrics. To solve this problem, we tested the amount of Fe in the fabric and then utilized H_2_C_2_O_4_ solution to wash the Fe from the fabric surface (Figure S6, the surface iron content of the fabrics treated with oxalic acid). As can be seen from Table 1, after dyeing, the K/S value show a slow decrease after oxalic acid pickling, and the value of L*, a* and b* change like this manner. Therein, L^*^ value represents the brightness of the fabric, the more positive the value, the brighter the fabric, and vice versa; a* value represents the red-green degree, the more positive the value, the redder the fabric, and vice versa; b* value represents the yellow-blue degree, the more positive the value, the yellower the fabric, and vice versa. Changes between L*, a* and b* indicate that after washing with oxalic acid, the surface of the fabric is brighter, its red light decreases, and its K/S value slightly decreases too. Further Scanning Electron Microscope (SEM) experiments also supported that the surface of fibers was smooth after washing with oxalic acid. (Figure S7, SEM experiments).

**Table 1:** Effect of oxalic acid post-treatment on dyeing effect.

Concentration of H_2_C_2_O_4_ g/L	L*	a*	b*	K/S
0 g/L	22.93	4.33	−18.1	16.49
2 g/L	23.28	4.19	−18.34	16.2
5 g/L	23.62	3.85	−18.07	16.03
10 g/L	24.88	2.55	−18.15	15.92
15 g/L	24.94	2.47	−18.38	15.87
20 g/L	24.78	2.45	−18.25	15.79

**Figure 6:**
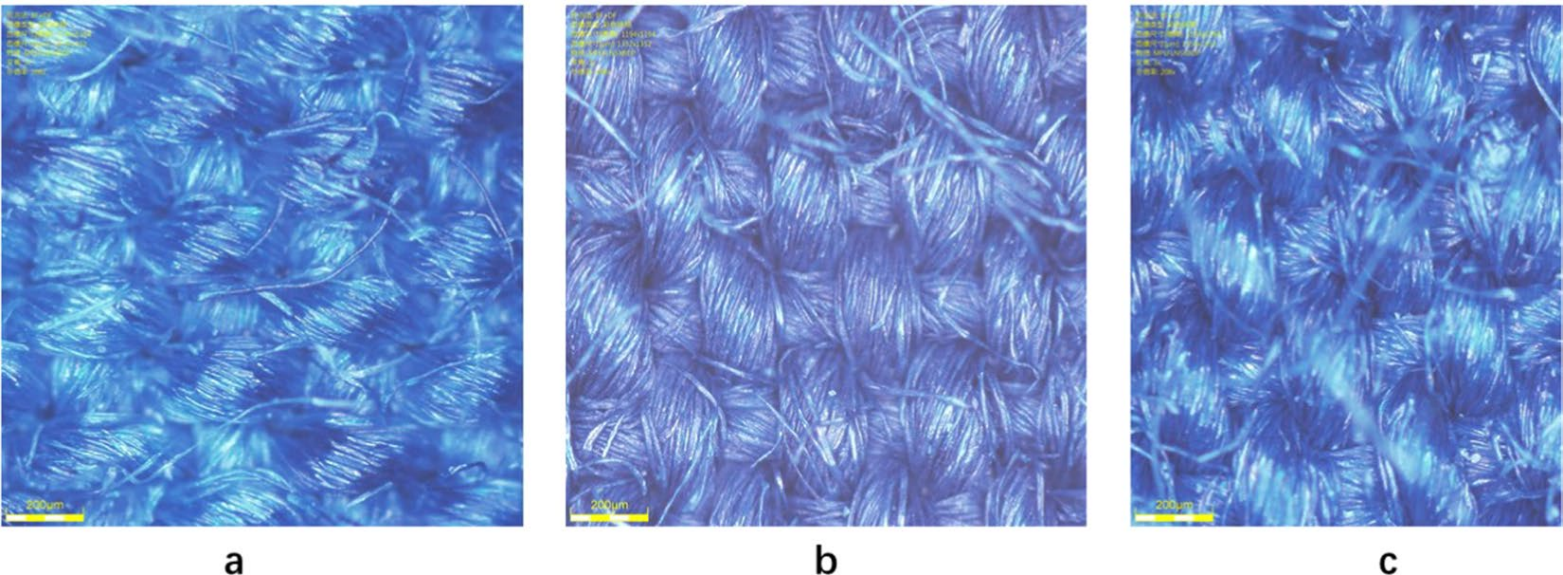
Dyeing process. (**a**) Traditional indigo dyed cloth using insurance powder as the reductant; (**b**) Electrochemical reduction dyed cloth without oxalic acid treatment; (**c**) Electrochemical reduction dyed cloth with oxalic acid treatment.

**Figure 7:**
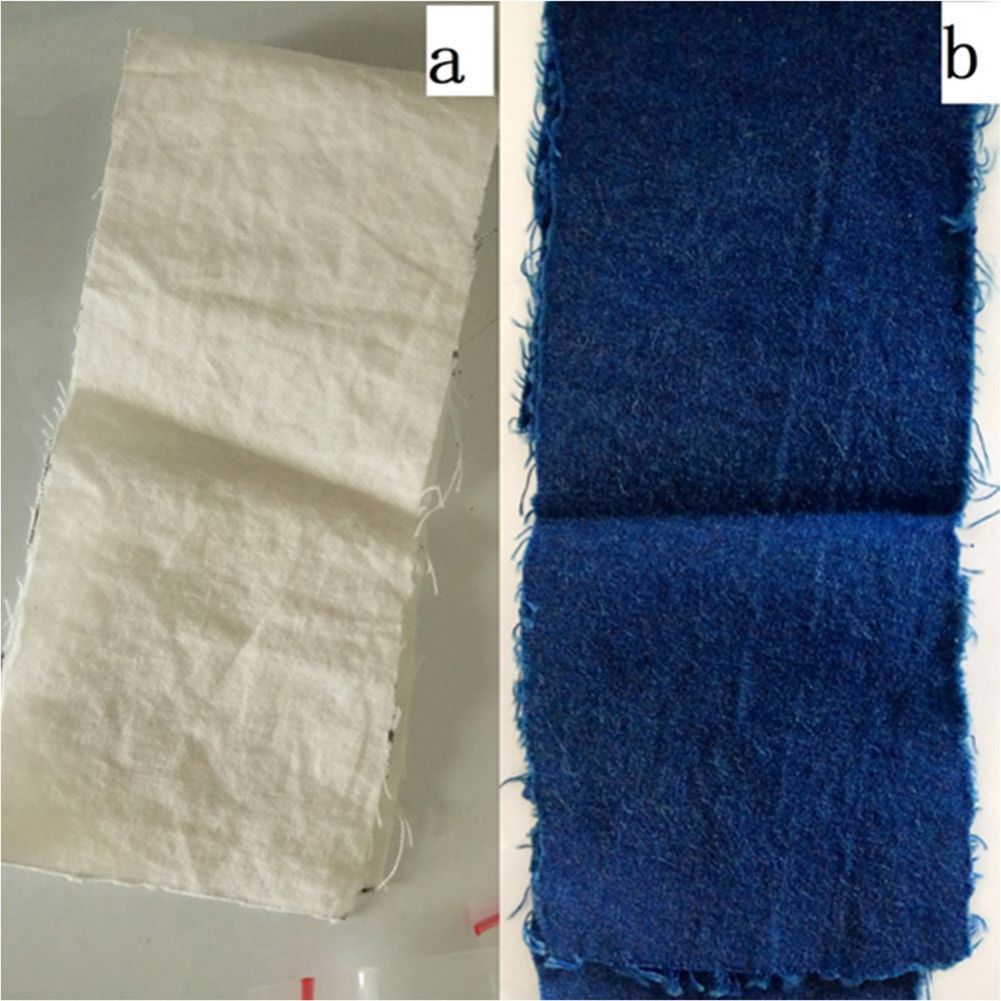
Dyed cloth. (**a**) undyed cloth. (**b**) Dyed cloth using electrocatalytic method.

**Figure 8:**
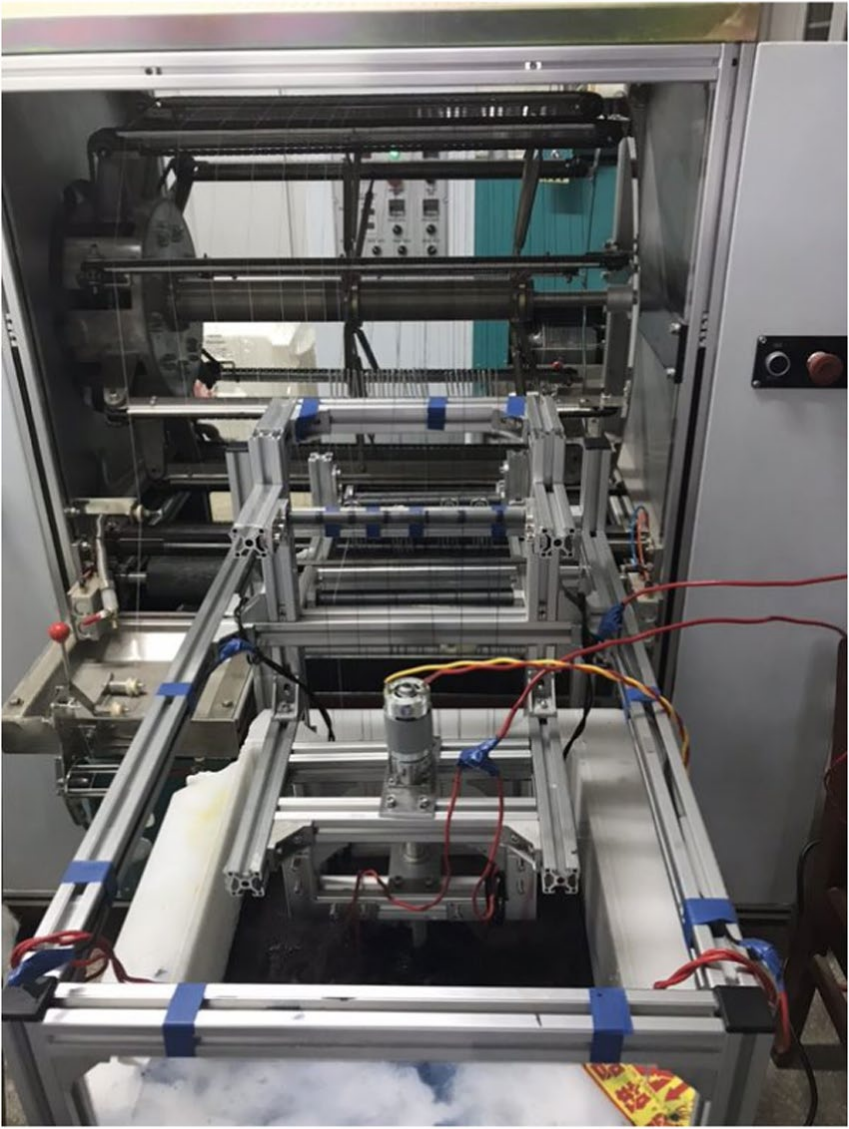
Electrochemical dyeing device in large scale.

After washing with H_2_C_2_O_4_ solution, further comparison was performed with optical microscope. Figure 6a was traditional indigo dyed cloth by using insurance powder as reducing agent. In the Fig. 6b, electrochemical reduction dyed cloth without oxalic acid treatment showed slightly red under the optical microscope, coming of the used ferric salt in electrochemical reduction method adsorbed on the fabric along with the indigo leuco and oxidized to iron oxide. By washing the electrochemical reduction dyed cloth with oxalic acid, the cloth can achieve the effect and standard of traditional dyed cloth (Figs. 6c and 7). This is because the ferric oxide and ferric hydroxide adhering to the original fabric reacted with oxalic acid, forming ferrous oxalate that is dissolved in water and can be washed off from the surface of the fiber. In order to explore the application of electrochemical reduction methods to actual production, a middle-scale electrochemical dyeing device was applied. As is shown in Fig. 8, indigos and intermedia were mixed in a dyeing tank, while cathode and anode were placed at the bottom of the tank. After indigo reduction, the yarn above the electrodes starts to work. During the experiment, reduction and dyeing work at the same time.

## Discussion

To sum up, we have reported a practical indirect process for the electro-chemical reduction of indigo. Using the developed device, we can realize the electro-reduction and dyeing at the same time. The iron-triethanolamine-calcium gluconate (Fe-TEOA-Ca) system features as the intermediate for reducing indigo in this process. The ultrasonic wave not only speeds up the currency efficiency, but also cleans up the cathodes. Washing with oxalic acid solution is found to be an effective way to get rid of iron salt on the fabric after dyeing. The dyed fabric could meet the level of color fastness in industry.

## Methods

Methods, including statements of data availability and experimental details and references, are available in the supplementary information.

### Dyeing experiment

Fabric was processed in scouring bath at 95 °C for 20 min before dyeing process, to avoid too much air carried by fabrics during the dyeing to produce bubbles. The scouring liquid was composed of 8 g/L NaOH and 5 g/L sodium metasilicate nonahydrate. Bath ratio = 1:100. Then the fabric was washed by deionized water three times and dried to ensure that its dyeing more thoroughly. In the dyeing experiment, dipping of the fabric in dye liquor for 30 s and airing for 2 min completes ‘1-dip 1-nip’ cycle. Samples for ‘6-dip 6-nip’ padding were passed through six such consecutive cycles, with a final airing for 3 min converting all reduced dye on the fabric to its oxidized state. The dyed samples were then subject to a cold rinse three times with deionized water and then dried at 100 °C for 2 min in a laboratory dryer. The dyeing experiment was performed at 25 °C.

## Supplementary information

SI.
