# Supplementary material for: Practical and environment-friendly indirect electrochemical reduction of indigo and dyeing
Source: Sci Rep. 2020 Mar 18;10:4927. doi: 10.1038/s41598-020-61795-5 (PMC7080776; doi:10.1038/s41598-020-61795-5)
Supplement: Supplementary file 1 — SI. [file 41598_2020_61795_MOESM1_ESM.docx]

**Supporting Information**

**Practical and environment-friendly indirect electrochemical reduction of indigo and dyeing**

Changhai Yi,*,^1^ Xiaodong Tan,^1,3^ Bihan Bie,^1^ Haitao Ma,^4^ Hong Yi*,^2^

^1^Science and Technology Institute, National Engineering Laboratory for Advanced Yarn and Fabric Formation and Clean Production, Wuhan Textile University, Wuhan, Hubei 430200, People’s Republic of China

^2^ College of Chemistry and Molecular Sciences, Wuhan University, Wuhan, Hubei 430072, People’s Republic of China

^3^ Department of Material Engineering, Faculty of Textile Engineering, Technical University of Liberec, Czech Republic

^4^ Shandong Ruyi Technology Group Company, Jining, Shandong, People’s Republic of China

**Experiment informations**

1. ***Chemicals and materials***

Indigo (98%), Ferric sulfate powder (Fe_,_ 21-23%), Oxalic acid (99%) and Calcium gluconate (99%) were analytical grade chemicals, supplied from the Aladdin Reagent Co., China. TEA, Sodium dithionite, Potassium ferricyanide, NaOH, Sodium metasilicate nonahydrate, KSCN, H_2_SO_4_ and H_2_O_2_ were analytical grade chemicals, supplied from the Sinopharm Chemical Reagent Co., China. Nickle electrode, Stainless steel electrode (4 $\times$ 5 cm^2^), glass carbon electrode (d = 3 mm), platinum electrode (0.5 $\times$ 0.5 cm^2^), and saturated calomel electrode (SCE) obtained from Gaossunion Co., China.

All the solutions were using Ultrapure water with 18 MΩ cm resistivity taken from Ulupure system.

Desized, scoured and bleached cotton fabric (plain weave, warp yarn density 30/cm, weft yarn density 30/cm) were used for dyeing experiment.

1. ***Polarization curve***

The working electrode was a nickel or stainless steel electrode, the auxiliary electrode was a platinum plate electrode and the reference electrode was a SCE. The test was carried out in a three-necked flask, and the SCE was placed in the salt bridge with the saturated potassium chloride solution. The initial potential was -0.2 V and the terminal potential was 0.2 V, the scanning rate was 0.5 mV/s, and the potential interval was 0.5 mV. The nickel electrode, stainless steel electrode, and platinum plate electrode were polished three times using metallographic paper until the surface was smooth before the test.

1. ***Current efficiency (CE) and Potentiometric titration***

The RE and CE were calculated by potentiometric titration using ZDJ-4A (Shanghai Lei-Ci CO.). 2 mL of the reduced solution was diluted to 10 mL with deionized water and then titrated that 4 g/L potassium ferricyanide was adopted as a titrant, and a platinum electrode as an indicator electrode. All the process of titration was executed under protection of nitrogen because the reduced solution would be oxidized in the air. The RE and CE were computed by the following two equation:

$$RE=\left[n\times C\times\Delta V\div\left(2ml\times2e\times C_{0}\right)\right]\times100\%$$

$$CE=\frac{RE\times C\times F\times n}{It}\times100\%$$

Where $n$ is the number of electrons needed to reduce indigo; $C$ is the molar concentration of the potassium ferricyanide; $\Delta V$ is the volume of potassium ferricyanide consumed; $C_{0}$ is the original molar concentration of indigo; $F$ is Faraday constant; $I$ is the intensity current (A); $\text{t}$ is the electrolysis time for indigo reduction (sec).

1. ***Cyclic voltammetry (CV) experiments***

CV experiments were conducted by Princeton-PMC electrochemical workstation and carried out at room temperature. The three-electrode system was uses as a CV experiments in which glass carbon electrode (d = 3 mm) was the working electrode, platinum electrode (0.5 $\times$ 0.5 cm^2^) was the counter electrode, SCE was the reference electrode. The mediator solution was deoxygenated for at least 30 min before CV experiments and under nitrogen protection to ensure that the redox peak would be recorded during CV scan properly. Glass carbon electrode and platinum electrode was also polished by metallographic sandpaper to get rid of surface dirt, and moved into ultrasonic bath for cleaning 3 min.

1. ***Dyeing experiment***

Fabric was processed in scouring bath at 95 ℃ for 20 min before dyeing process, to avoid too much air carried by fabrics in the dyeing process to produce bubbles. The scouring liquid was composed of 8 g/L NaOH and 5 g/L sodium metasilicate nonahydrate. Bath ratio = 1:100. Then the fabric was washed by deionized water three times and dried to ensure that its dyeing more fully. In the dyeing experiment, dipping of the fabric in dye liquor for 30 s and airing for 2 min completes ‘1-dip 1-nip’ cycle. Samples for ‘6-dip 6-nip’ padding were passed through six such consecutive cycles, with a final airing for 3 min converting all reduced dye on the fabric to its oxidized state. The dyed samples were then subject to a cold rinse three times with deionized water and then dried at 100 ℃ for 2 min in a laboratory dryer. The dyeing temperature is 25 ℃.

1. ***Dyeing sample evaluations***

Five points was selected randomly on the sample and measured respectively. The result of dyeing experiment was characterized by color measurement in the form of K/S using Colori7 spectrophotometer system supplied from the X-rite Co., American.

1. ***Estimation of iron in the dyed samples***
   1. ***Preparation of solution for calibration***

Ferric ammonium sulphate (864 mg) was dissolved in an amount of water, the volume of which was adjusted to 1 l after dissolution (concentration of ferric iron in this solution was 0.1 mg/ml). To 100 ml of this solution, 10 ml concentrated hydrochloric acid, followed by 10 ml of 30% potassium thiocyanate were added (final solution was a blood red color) and diluted to 1 l (the concentration of ferric iron had now been reduced to 0.01 mg/ml). Ten samples of this solution each of varying amounts (10, 20, 30, 40, 50, 60, 70, 80, 90 and 100 ml) were then pipetted into ten different 100 ml capacity volumetric flasks and the volume in each was made up to 100 ml with water (as necessary).

- 1. ***Preparation of calibration curve***

Optical densities of the red solutions of varying concentrations were measured on a UV-7504 visible spectrophotometer at 470 nm against a blank solution containing 10 ml concentrated hydrochloric acid and 10 ml 30% potassium thiocyanate made up to a volume of 1 l with water. The optical density values when plotted against concentration of ferric iron, showed a linear relationship and thus this was considered as the standard calibration curve.

- 1. ***Estimation of ferric iron***

A known weight of dyed sample was taken and treated in 10% hydrochloric acid solution for 1 min at 80–90 °C. The extract was collected and to it 10 ml concentrated

hydrochloric acid and 10 ml 30% potassium thiocyanate were added, diluted to 1 l and the optical density of this solution was then measured. The concentration of ferric iron was calculated from the calibration curve and then was calculated for 100 g cotton.

1. ***Test of SEM***

After spraying by golds, dyed samples and electrodes were laid on EVO 18 scanning electron microscope for observing.

Table S1. Different factors and levels of L_9_ (3^4^) orthogonal experiment

| Level | Factor | | | |
| --- | --- | --- | --- | --- |
|  | A | B | C | D |
|  | Fe_2_(SO_4_)_3_ | NaOH | TEOA | Calcium gluconate |
| 1 | 5 g/L | 5 g/L | 15 g/L | 5 g/L |
| 2 | 10 g/L | 15 g/L | 30 g/L | 10 g/L |
| 3 | 15 g/L | 25 g/L | 45 g/L | 15 g/L |

Table S2. Results analysis of L_9_ (3^4^) orthogonal experiment

| No. | Factor | | | | |
| --- | --- | --- | --- | --- | --- |
|  | A | B | C | D | CE (%) |
| 1 | 1 | 1 | 1 | 1 | 32.6 |
| 2 | 1 | 2 | 2 | 2 | 68.1 |
| 3 | 1 | 3 | 3 | 3 | 62.3 |
| 4 | 2 | 1 | 2 | 3 | 37.8 |
| 5 | 2 | 2 | 3 | 1 | 69.3 |
| 6 | 2 | 3 | 1 | 2 | 64.7 |
| 7 | 3 | 1 | 3 | 2 | 35.1 |
| 8 | 3 | 2 | 1 | 3 | 45.7 |
| 9 | 3 | 3 | 2 | 1 | 70.1 |
| X_1_^*^ | 54.3 | 35.2 | 47.7 | 57.3 |  |
| X_2_^*^ | 57.3 | 61 | 58.7 | 56 |  |
| X_3_^*^ | 50.3 | 65.7 | 55.7 | 48.5 |  |
| R | 7 | 30.5 | 11 | 8.8 |  |

X_i_^*^=$\frac{1}{n}\sum X_{i}^{A,B,C,D}$ R=X_max_−X_min_

In the electrolysis experiment, many factors will have effects on CE. So, a different factors and levels of L_9_ (3^4^) orthogonal experiment are carried out. In Table S1, Fe_2_(SO_4_)_3_, NaOH, TEOA and Calcium gluconate were selected as four main factors to be researched.

As can be seen from Table S2, the concentration of NaOH has the greatest influence on the electrolysis experiment, while TEOA and Calcium gluconate follow behind. Because reducing indigos depends on the PH environment.


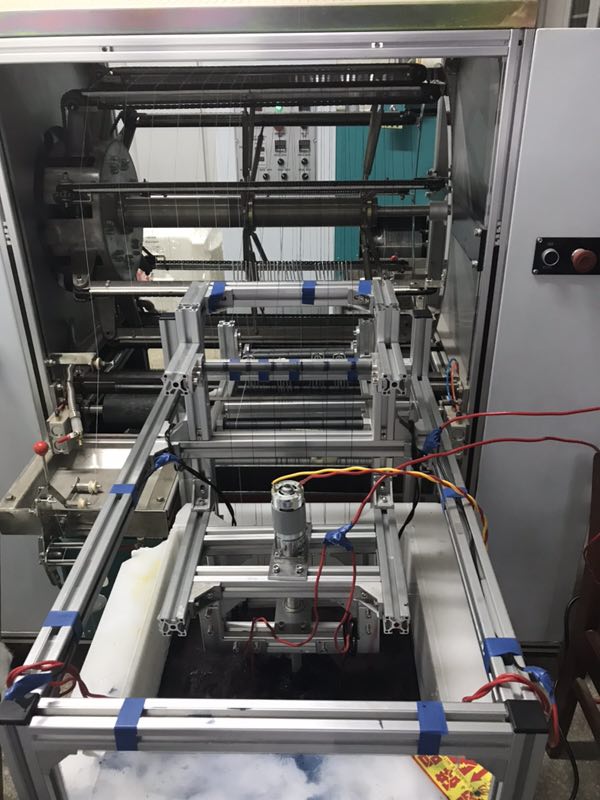


Figure S1. Electrochemical dyeing device

As is shown in Figure S1, we tried to experiment on a middle-scale device. By applying the electrochemical reduction method, it achieved reduction of indigo in high capacity and dyed yarns simultaneously. This process is almost as the same as the process of factory. Also, if we want to apply this method in the factory production line, there are still some details that we have to solve.


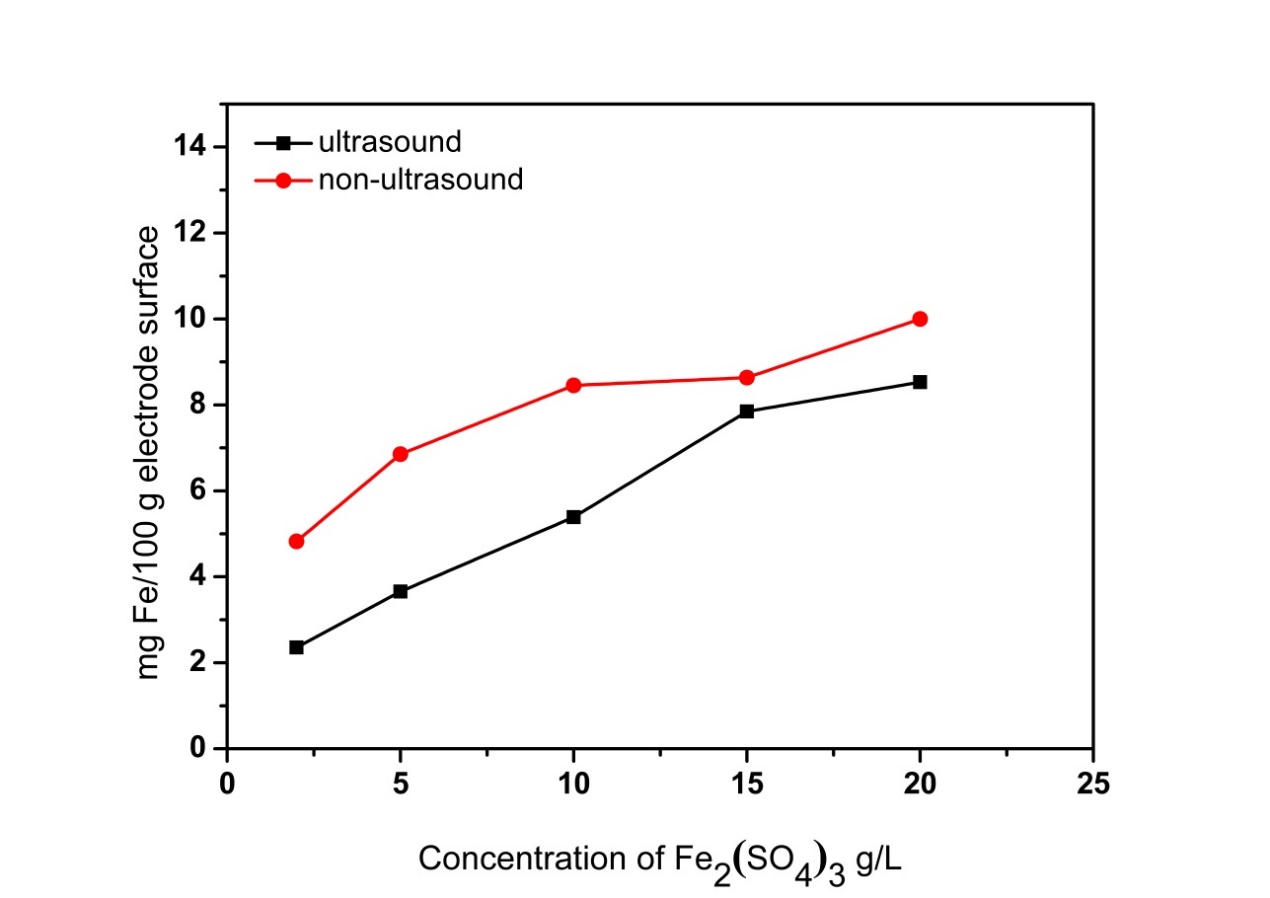


Figure S2. Iron deposition on the cathode surface

It can be seen from the Figure S2 that there are many Fe ions deposit on the surface of cathode with the increasing concentration of ferric sulfate under electrochemical reaction, but the content of Fe ions of electrode with ultrasound is relatively lower than that of electrode without ultrasound.


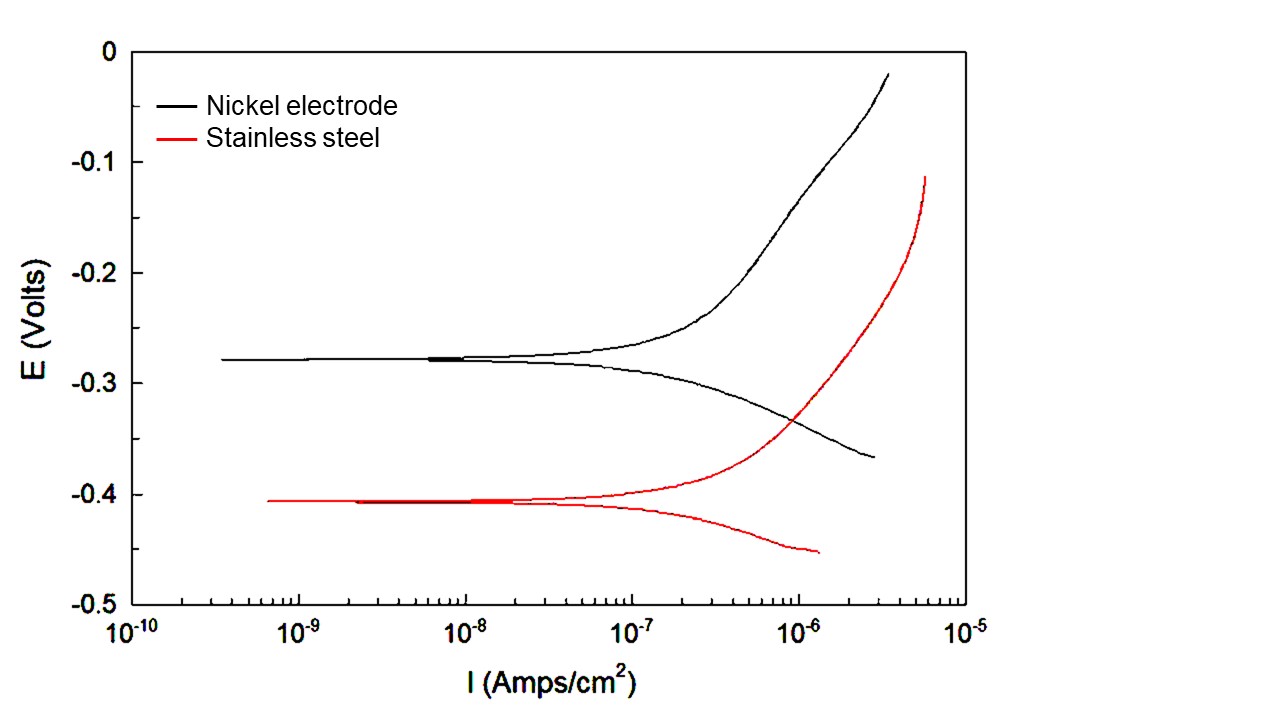


Figure S3. Polarization curve of different electrode in Ca2+-Fe3+-TEA mediator solution

As seen from the Figure S3, it is obvious that the potential of Fe-TEA-Ca indigo solution is higher than that of Fe-TEA indigo solution, and the polarization current of Fe-TEA-Ca indigo solution is smaller, which indicates that the corrosion degree of dinuclear system indigo solution decreases and the corrosion rate slows down.

Also, from the investigation of current efficiency between nickel and stainless steel electrodes, we found that in low concentration of indigos both nickel and stainless steel electrodes have good electrochemical properties. But when adding more indigos, the CE of stainless steel electrodes declined rapidly, while nickel electrodes declined slowly.


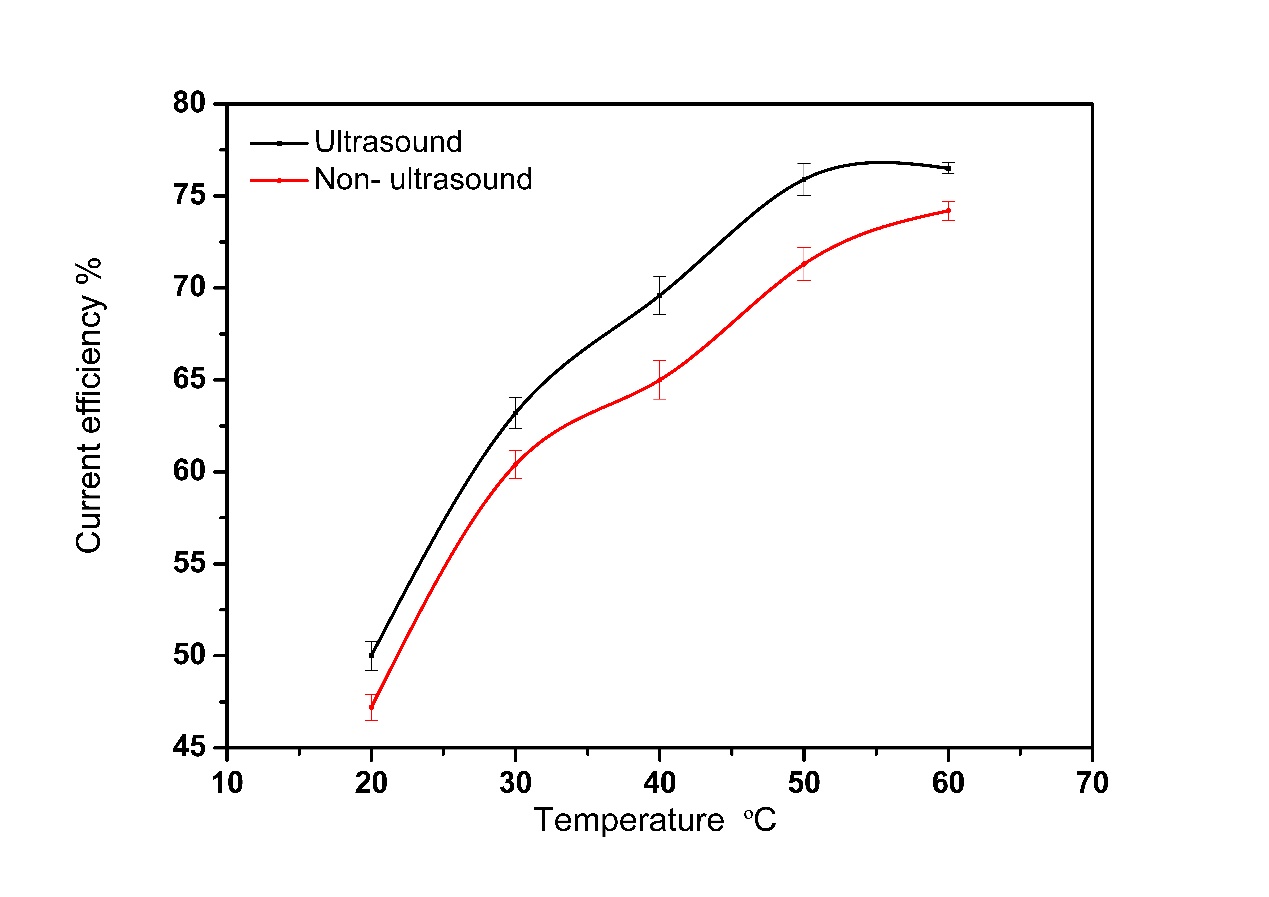


Figure S4. Investigation of pool temperature

As shown in the Figure S4 above, with the increase of temperature, reduction efficiency and current efficiency are gradually improved. However, when the temperature rises to 60℃, the solution evaporates, which makes the solution concentration getting unstable. In conclusion, the optimum reduction temperature should be 50℃.


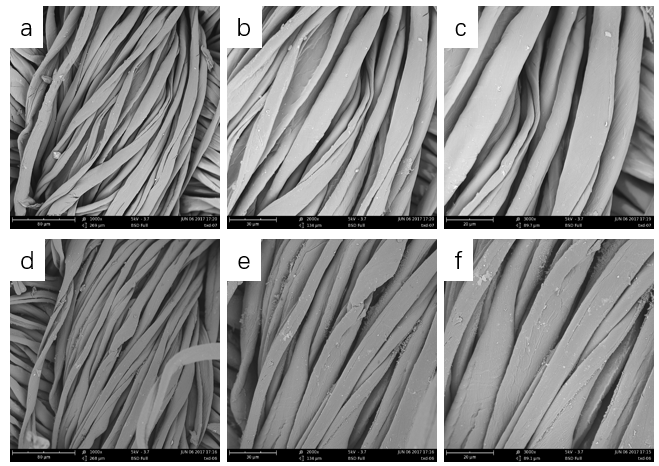


Figure S5. Scanning image of pure white cotton cloth and traditional indigo dyed cloth

This Figure S5 is the scanning image of pure white cotton cloth and traditional indigo dyed cloth under 1000, 2000 and 3000 times SEM. As shown in Figure , compared with the traditional indigo dyed fabric, the pure white cotton fabric without dyeing is smoother and has less impurities. In traditional indigo dyeing cloth, there are a few granular substances.


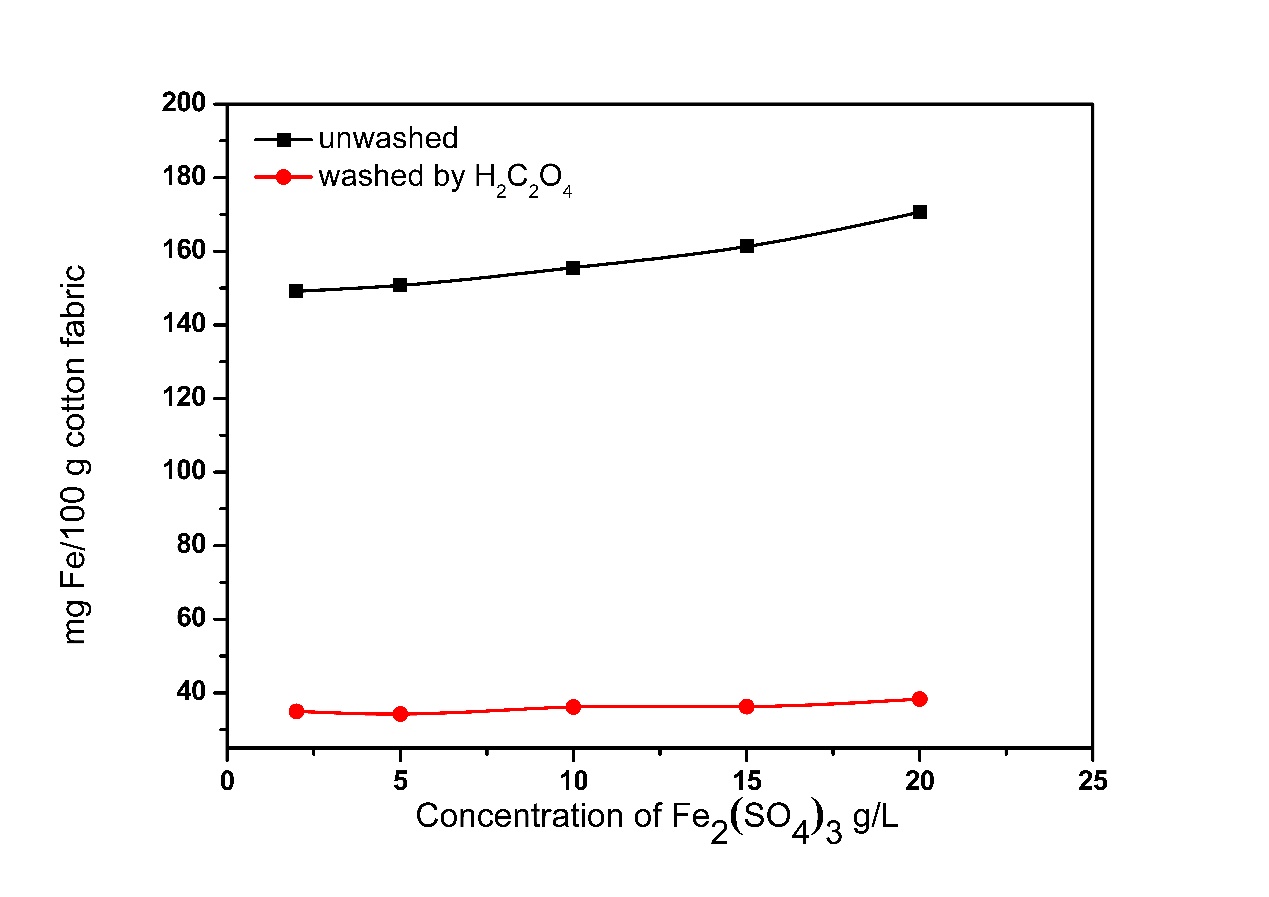


Figure S6. Surface iron content of the fabrics treated with oxalic acid

From the Figure S6 we can see that the surface iron content of the fabrics treated with oxalic acid decreases significantly, which indicates that after oxalic acid treatment, the ferric ions adhering to the surface of the fabrics have been washed off, but a small amount of iron are still left on the surface of the fabrics.

In addition, we have tried the dilute hydrochloric acid, dilute sulfuric acid and citric acid. Dilute hydrochloric acid and dilute sulfuric acid have good performance as the same as the oxalic acid, while citric acids got poor performance. Those acids can remove the Fe ions, but dilute hydrochloric acids and dilute sulfuric acids cost higher. So, we choose oxalic acid as our main material to remove ferric ions.


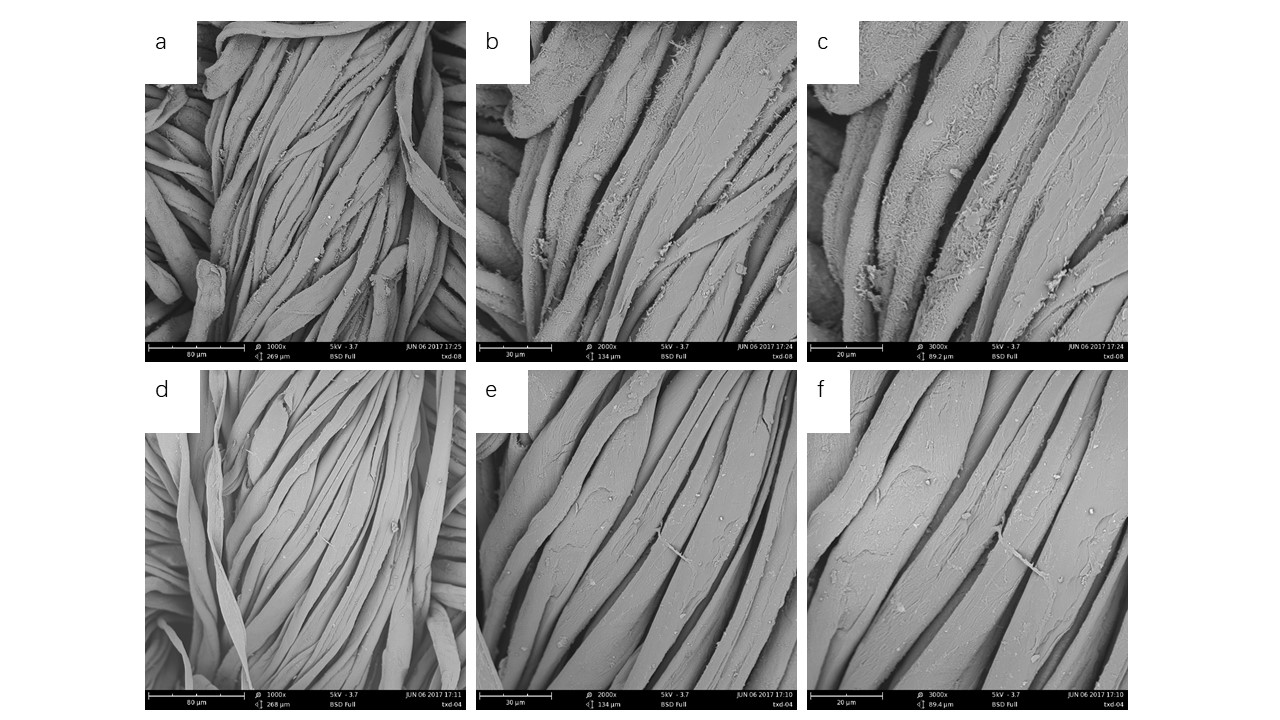


Figure S7. SEM experiments

This Figure S7 is a comparison of electrochemical reduction dyed fabrics before and after being washed with oxalic acid. It can be clearly seen that there are many impurities on the surface of fabric fibers which have not been washed by oxalic acid, and the surface is rough with many particulate matter on the surface of the fibers, while the surface of fibers washed with oxalic acid is smooth.
